# Protein Kinase D2 Protects against Acute Colitis Induced by Dextran Sulfate Sodium in Mice

**DOI:** 10.1038/srep34079

**Published:** 2016-09-23

**Authors:** Jing Xiong, Ming-feng Zhou, Ya-dong Wang, Li-ping Chen, Wan-fu Xu, Yao-dong Wang, Fan Deng, Si-de Liu

**Affiliations:** 1 Guangdong Provincial Key Laboratory of Gastroenterology, Department of Gastroenterology, Nanfang Hospital, Southern Medical University, Guangzhou 510515, China; 2 Department of Cell Biology, School of Basic Medical Sciences, Southern Medical University, Guangzhou 510515, China

## Abstract

Inflammatory bowel disease is characterized by dysregulation of the mucosal immune system resulting from impaired intestinal epithelial barrier function. Protein kinase D2 has been implicated in the regulation of immune responses. The present study was to define PKD2 might affect murine colitis. Colitis was induced in wild-type mice (PKD2^WT/WT^) and PKD2 catalytic activity deficient mice (PKD2^SSAA/SSAA^) with dextran sulfate sodium. PKD2^SSAA^-knockin mice displayed catalytic activity deficiency and increased susceptibility to DSS-induced colitis with enhanced weight loss, colonic inflammation compared with PKD2^WT/WT^ mice. Furthermore, crucial inflammatory cytokines mRNA levels in PKD2^SSAA^-knockin mice were higher than controls accompanied with down-regulation of ZO-1, MUC2 and intestinal barrier dysfunction. However, there were no differences in the proliferation or apoptosis of intestinal epithelial cells in PKD2^SSAA^-knockin mice compared with wild-type controls. In addition, PKD2 expression was repressed in patients with IBD compared with healthy controls. These studies suggested that activation of PKD2 in the colonic epithelium microenvironment may contribute to protect against DSS-induced colitis through regulation of intestinal mucosal immunity and barrier function.

Inflammatory bowel disease (IBD), characterized by chronic and recurrent intestinal inflammation, affects over 3.6 million people worldwide^1^ and is associated with high economic costs in terms of patients, healthcare and society^2^. Although the precise pathogenesis underlying IBD is still poorly understood, it is widely accepted that a complex interplay among genetic, environmental and immunological factors contributes to the development of IBD^3^,^4^. Genome wide association studies (GWAS) have revealed at least 163 host susceptibility loci associated with risk of IBD^5^, further highlighting the role of genes in this disease. Identification of additional susceptibility gene and their molecular functions is necessary to explore new therapeutic targets for IBD.

The intestinal epithelium forms a protective barrier to prevent permeation of luminal microbiota and foreign antigens into mucosal tissues^6^. This barrier mainly consists of the mucus layer, tight junctions (TJs) and intestinal epithelial cells (IECs), whose integrity was largely mediated by tight junction function^7^. In fact, barrier defect with altered expression of TJs and increased intestinal permeability are closely linked to the course of IBD^8^,^9^,^10^. In addition, IECs are also actively involved in the innate immune response as many epithelial cells secrete cytokines and chemokines.

Protein kinase D2 (PKD2) is a member of a new family of serine/threonine protein kinases composed of 2 other different isoforms, PKD1 and PKD3^11^. PKD has been implicated in diverse biological processes, including epithelial barrier function and inflammation^12^,^13^,^14^. PKD, especially PKD3, disrupted airway epithelial barrier integrity mainly by reducing claudin-1 expression^12^. Another study reported that CID755673, a kind of PKD specific inhibitors, alleviated of necrosis and severity of acute pancreatitis in mice effectively^15^. Nonetheless, the role of PKD2 in IBD remains undetermined.

In the present study, we showed that PKD2 enzymatic deficiency mice exhibit elevated susceptibility to dextran sulfate sodium (DSS)-induced colitis compared with wild-type control, up-regulated expression of crucial pro-inflammatory cytokines and disrupted epithelial barrier function. However, no differences in the proliferation or apoptosis of intestinal epithelial cells in mice were observed. Furthermore, decreased epithelial expression of PKD2 was found in patients with IBD. Thus, our data firstly demonstrate a protective role for PKD2 in intestinal inflammation.

## Results

### Genotypes and Phenotypes of Mutant Mice

**Figure 1:**
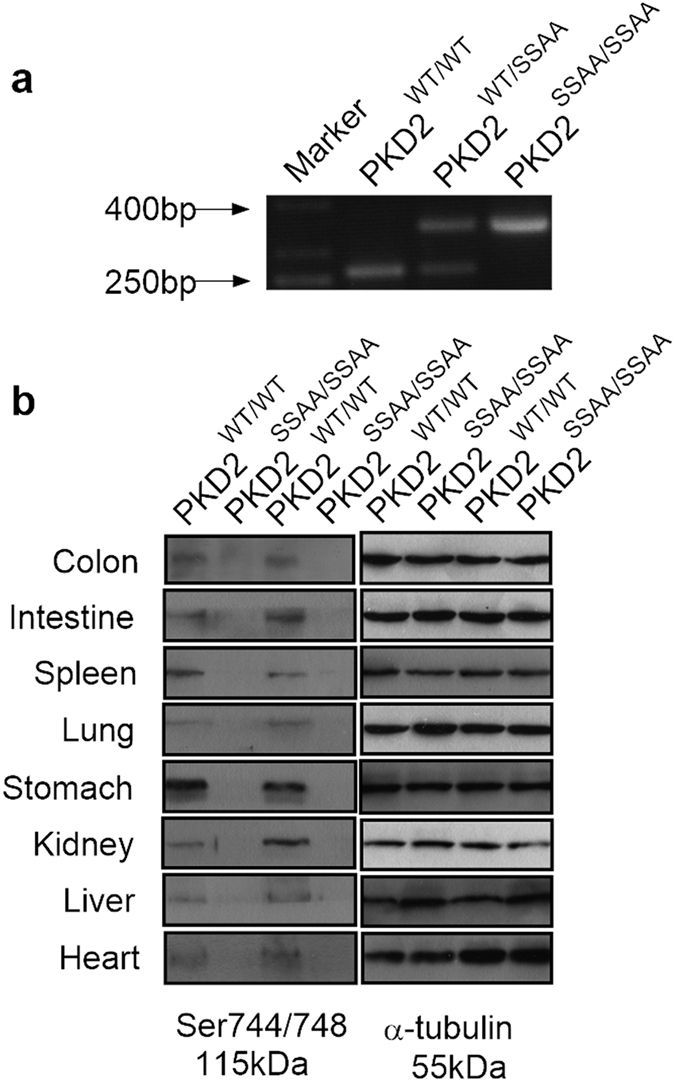
Identification of PKD2 catalytic activity deficiency mice. (**a**) Genotypes of PKD2^SSAA/SSAA^ mice were determined by PCR amplification of genomic DNA. The wild-type allele generates a 236 bp product, whereas the knockin allele generates a 344 bp product. (**b**) Western blot analysis of p-PKD744/748 expression in organs from wild-type and PKD2^SSAA/SSAA^ mice.

To study the function of PKD2 in UC, we obtained homozygous PKD2 catalytic activity deficiency mice which PKD2 S707A and S711A mutations were knocked into the wild-type *Prkd2* locus by mating mutant heterozygotes^16^. Genotypes of PKD2^SSAA^ mutant mice were determined by PCR amplification of genomic DNA. As expected, a 236-bp DNA product was amplified from wild-type mice, whereas PKD2^SSAA^ knockin mice generated a 344-bp product (Fig. 1a). Further western analysis of the activated or phosphorylated proteins level of PKD2 in different tissues of mice revealed that there was no phosphorylation on Ser744 and Ser748 site of activation loop of PKD2 in different tissues from PKD2^SSAA/SSAA^ mutant mice compared with PKD2 wild type mice, demonstrating that the presence of homozygous PKD2 catalytic activity deficiency in PKD2^SSAA/SSAA^ mutant mice (Fig. 1b).

### PKD2 catalytic activity deficiency exacerbates disease severity in DSS-induced colitis

**Figure 2:**
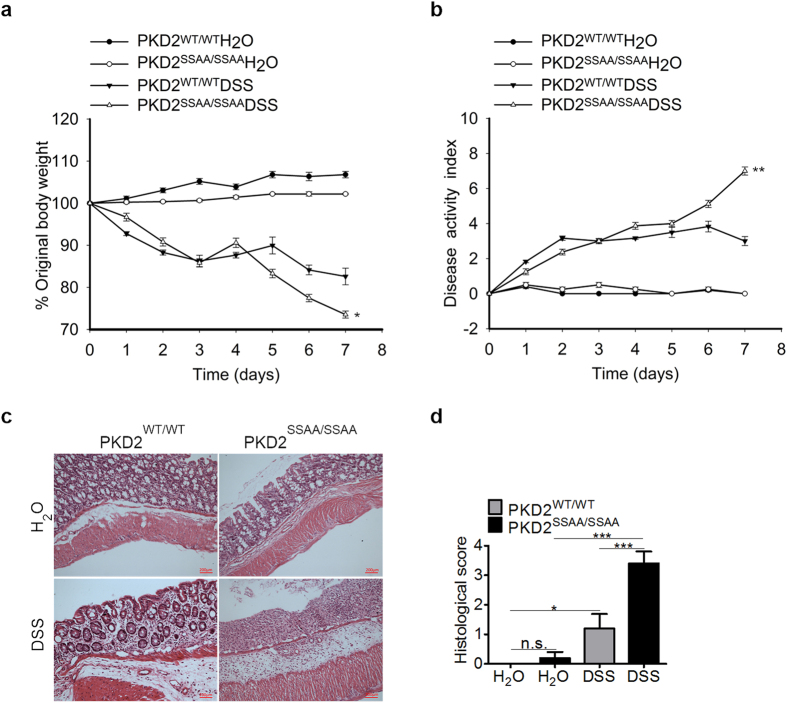
Disease activity in PKD2^SSAA/SSAA^ mice during DSS-colitis. Gender-, age- and weight-matched mice with PKD2 mutant (PKD2^SSAA/SSAA^) and their wild-type controls (PKD2^WT/WT^) were exposed to DSS (5%) for 7 days, followed by sacrifice and harvesting of the whole colon (n = 8). (**a**) Daily weight measurements were obtained for each group of mice. *p < 0.05 as measured by ANOVA for Repeated Measures. (**b**) Daily disease activity measurements encompassing weight, stool consistency and presence of blood were assessed for each group of mice. **p < 0.01 as measured by ANOVA for Repeated Measures. (**c**) Representative histological sections from whole colon of PKD2^SSAA/SSAA^ or PKD2^WT/WT^ mice harvested following 7 days of DSS or water. Bar represents 200 μm. Images acquired at 200×. (**d**) Blinded histological scoring of colonic tissue post DSS. All results are representative of three independent experiments with five mice per group and are displayed as mean ± SEM. ***p < 0.001 as measured by one way-ANOVA among four groups.

Previous study have demonstrated that PKD2 was the primary isoform of PKD expressed in murine lymphocytes^16^, thus prompting our investigation the role of PKD2 catalytic activity deficiency in experimental murine colitis. After monitoring clinical development of colitis for 7 days, mice were euthanized and colonic parameters further quantified. We observed that DSS treatment resulted in significantly increased weight loss (Fig. 2a) and disease activity index (Fig. 2b) in PKD2^SSAA/SSAA^ mice compared with wild-type mice. In accordance, histopathological analysis of H&E-staining colon section showed that marked inflammation and immune cells influx and the overall histological score in PKD2^SSAA/SSAA^ mice were dramatically increased by DSS induction (Fig. 2c,d). Together, these data indicated that PKD2 catalytic activity deficiency is detrimental to the development of DSS-induced colitis in mice.

### PKD2 has a protective effect on epithelial barrier function *in vivo* or *vitro*

**Figure 3:**
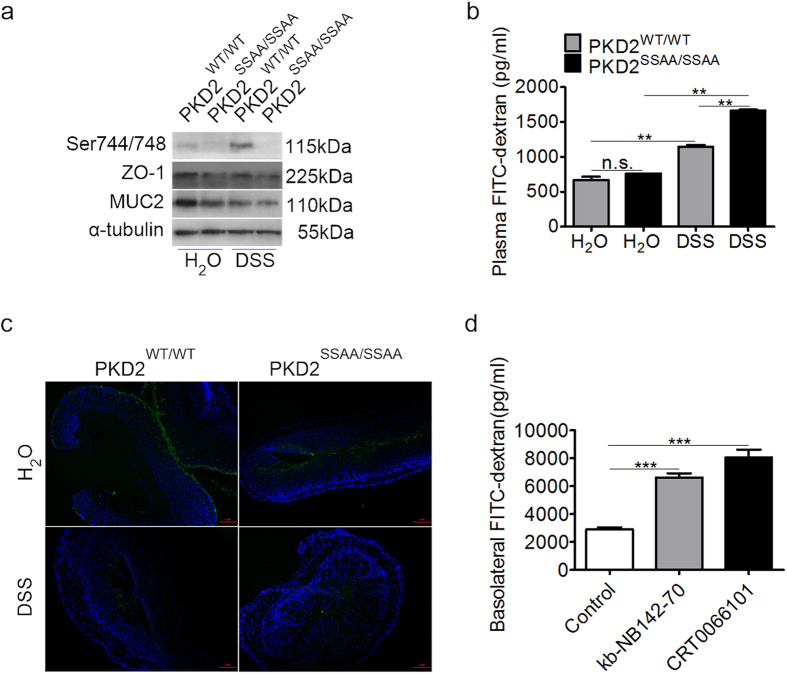
PKD2 has a protective effect on epithelial barrier function *in vivo* or *vitro*. (**a**) PKD2 knockin mutant *in vivo* decreased the expression of ZO-1and MUC2 in colonic mucosa of PKD2^SSAA/SSAA^ and PKD2^WT/WT^ mice. (**b**) Plasma fluorescene was measured following oral gavage of mice with FITC-dextran (4 kDa) exposed to DSS. ***p < 0.001 as measured by one way-ANOVA among four groups. (**c**) Representative images (50×) of immunofluorescence staining performed on frozen sections of colon from DSS-treated mice exposed to orally administered FITC-dextran (4 kDa) shows FITC permeation into lumen (green) is enhanced in PKD2^SSAA/SSAA^ mice treated with DSS compared with wild-type controls. Nuclei are stained with DAPI (blue). Bar represents 2 μm. (**d**) PKD specific inhibitors increases permeability of Caco-2 cells to FITC-dextran. Results represent three independent experiments with two mice per group.

It has been widely accepted that DSS-induced colitis is characterized by defect of the colonic epithelial barrier integrity whereby increasing intestinal permeability^17^. To assess the role of PKD2 in epithelial barrier function, we first analyzed the expression of ZO-1 and MUC2 in colon of mice with DSS-induced colitis. As shown in Fig. 3a, PKD2 catalytic activity deficiency caused a significant decrease in ZO-1 and MUC2 protein level. Defect of TJs may lead to dysfunction of the epithelial barrier. We then tested this by oral administration of FITC-dextran in mice exposed to DSS. PKD2^SSAA/SSAA^ mice had more FITC-dextran in plasma than wild-type controls did (Fig. 3b). Furthermore, fluorescent microphotography showed retention of FITC at the surface of the epithelial barrier in wild-type mice, whereas more FITC-dextran passed through the colonic epithelia barrier of PKD2^SSAA/SSAA^ mice (Fig. 3c).

To further confirm that PKD2 regulates intestinal barrier function, we also did *in vitro* experiments using Caco-2 cells monolayers, a well-established model system to study human intestinal barrier^18^. As expected, we found that the trans-epithelial flux of FITC-dextran was markedly increased in polarized Caco-2 cells after treatment with PKD specific inhibitors kb-NB142-70 or CRT0066101, indicating an enhanced paracellular permeability in response to PKD inhibition (Fig. 3d). To determine whether increased FITC-dextran permeability induced by PKD inhibitors were due to TJs, the expression of ZO-1 and Occludin from the treated Caco-2 and NCM460 cells were assessed. As shown in Supplementary Fig. S1, PKD activity inhibition triggered by kb-NB142-70 or CRT0066101 inhibitors caused the decreased expression of TJs and MUC2 in a dose-dependent manner in both NCM1460 and Caco-2 epithelial cells. Similarly, we also found that silencing of PKD2 by siRNA transient transfection led to reduction of TJs (Fig. S1a) and MUC2 (Fig. S1b) of intestinal epithelial cell monolayers as well. Altogether, these data suggested that PKD2 may protect against barrier dysfunction intestinal epithelial cells through regulation of TJs expression.

### PKD2 enzymatic deficiency has no effect on the proliferation or apoptosis of intestinal epithelial cells

**Figure 4:**
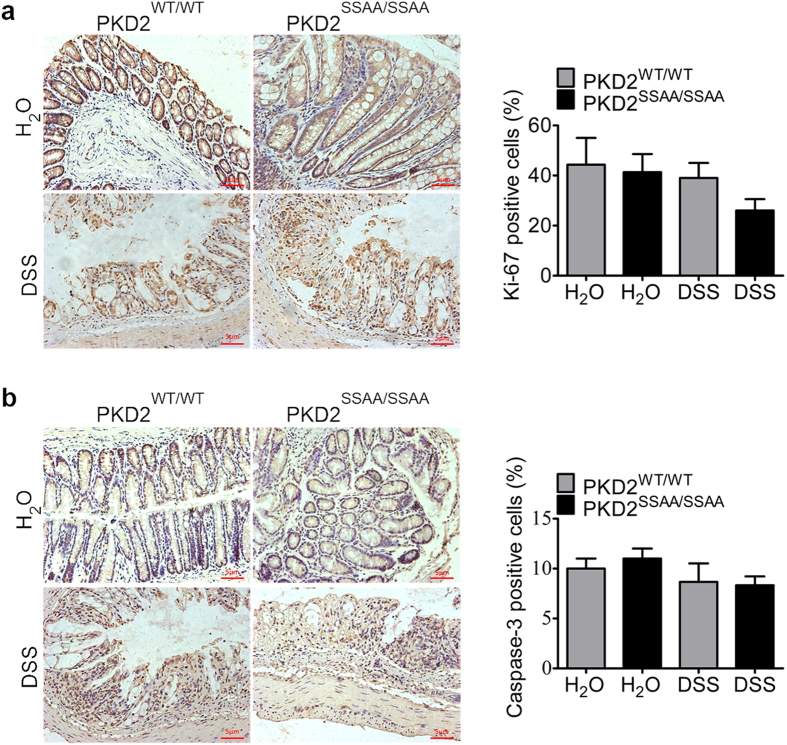
PKD2 enzymatic deficiency had no effect on the proliferation or apoptosis of intestinal epithelial cells in mice exposed to DSS. (**a**) Representative 200× immunohistochemical images (left) and quantification of Ki-67^+^ proliferating intestinal epithelial cells (right) in wild-type and PKD2^SSAA/SSAA^ mice. Bar represents 5 μm. (**b**) Representative 200× immunohistochemical images (left) and quantification of cleaved Caspase-3^+^ apoptotic intestinal epithelial cells (right) in wild-type and PKD2^SSAA/SSAA^ mice. Bar represents 5 μm. Results are representative of three separate experiments with three mice per group. All p > 0.05 as measured by one way-ANOVA among four groups.

Intestinal barrier function does not only correlated with tight junctions, but also related with epithelial integrity and apoptosis. Then we verified whether the barrier dysfunction observed in PKD2^SSAA/SSAA^ mice was probably due to the decreased proliferation or increased apoptosis of intestinal epithelial cells. Colonic staining for Ki-67 (proliferation marker) and cleaved Caspase-3 (apoptosis marker) showed that there was no difference for Ki-67 and Caspase-3 expression between wild-type and PKD2^SSAA/SSAA^ mice either with or without DSS treatment, indicating no difference in proliferation or apoptosis among groups (Fig. 4a,b). Collectively, the above findings indicated that the barrier-disruptive effects of PKD2 enzymatic deficiency are associated with tight junctions but not with decreased proliferation or increased apoptosis of intestinal epithelial cells.

### Inflammatory genes are upregulated in PKD2 enzymatic deficiency mice and in epithelial cells

**Figure 5:**
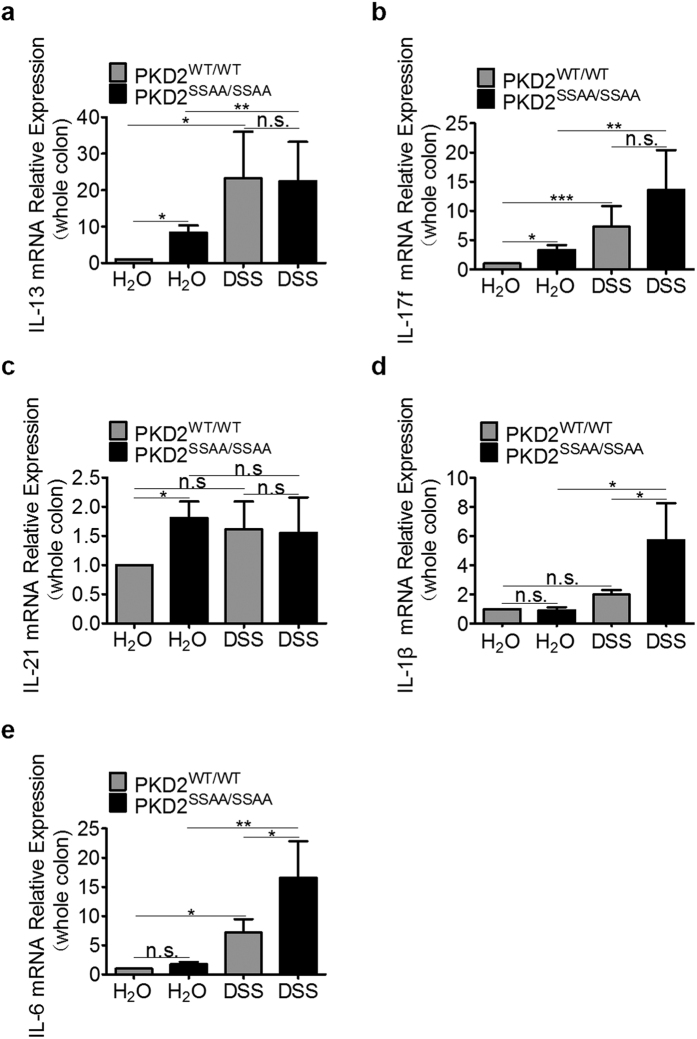
Colonic inflammatory cytokines increased in PKD2^SSAA/SSAA^ mice. Gender-, age- and weight-matched mice with PKD2 mutant (PKD2^SSAA/SSAA^) and their wild-type controls (PKD2^WT/WT^) were exposed to DSS (5%) for 7 days, followed by sacrifice and harvesting of the whole colon. Following whole colon harvest, total RNA was extracted, and the transcript levels of interleukin (IL) 13 (**a**), IL-17f (**b**), IL-21 (**c**), IL-1β (**d**) and IL-6 (**e**) were determined by real-time reverse transcriptase polymerase chain reaction (RT-PCR). Gene expression was calculated relative to GAPDH and expressed as fold change relative to water-exposed PKD2^WT/WT^ mice. Results represent three independent experiments with three mice per group (RT-PCR). All p < 0.05 as measured by analysis of variance.

Since dysregulation of mucosal immunity have been shown to play a crucial role in the pathogenesis of IBD, we first performed mRNA analysis of cytokines from colons of mice. We found that cytokine IL-13 (Fig. 5a), IL-17f (Fig. 5b) and IL-21 (Fig. 5c), which were known to be involved in IBD, were significantly upregulated in untreated PKD2 enzymatic deficiency mice compared with wild-type controls. Furthermore, the expression level of IL-1β (Fig. 5d) and IL-6 (Fig. 5e) were increased after DSS treatment. These data suggested that PKD2 enzymatic deficiency increases expression of several vital inflammatory genes contributing to the promotion of DSS-induced murine colitis.

**Figure 6:**
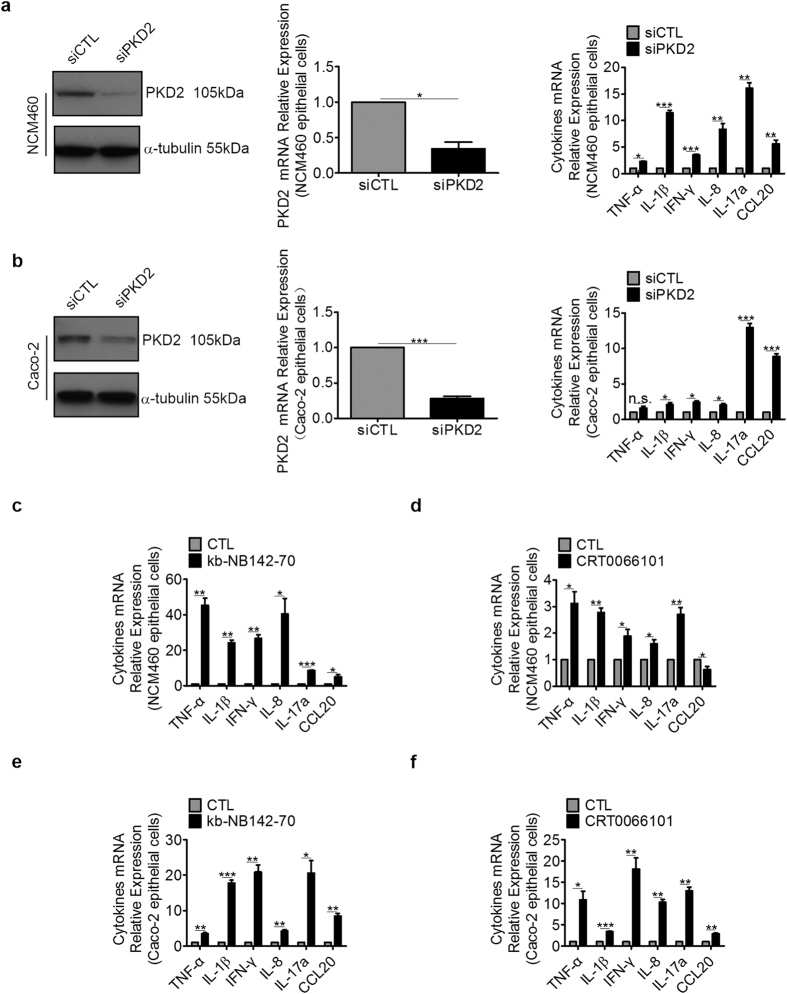
PKD2 decreased the mRNA levels of inflammatory cytokines. NCM460 cells or Caco-2 cells were transiently transfected with PKD2 siRNA (siPKD2). At 60 hours post-transfection, whole cell lysates were separated by SDS-PAGE and immunoblotted. Meanwhile total RNA was extracted for analysis of PKD2 and multiple cytokines (**a,b**) by RT-PCR. Results are mean ± SEM for triplicate samples and are representative of three separate experiments. NCM460 cells or Caco-2 cells were treated with 10 μM PKD inhibitor kb-NB142-70 or CRT0066101. At 24 hours, total RNA was extracted for analysis of multiple cytokines (**c–f**) by RT-PCR. Results are mean ± SEM for triplicate samples and are representative of three separate experiments.

In addition, we analyzed the protein expression of the 3 different PKD isoforms in 2 normal epithelial colon cell lines (NCM460 and IEC-6) and 2 colorectal cancer cell lines (Caco-2 and HT-29). As shown in Supplementary Fig. S2, PKD1 was only expressed in IEC-6 cell lines and almost not detected in other 3 cell lines. In contrast, PKD2 was expressed in all cell lines except in IEC-6 cell lines, while PKD3 was expressed in all cell lines. Moreover, PKD2 expression at the protein level in the epithelial colon cell lines was higher than other PKD isoforms revealing that PKD2 being the dominantly expressed isoform. We then performed gene knock-down experiments in NCM460 cells using PKD2-specific siRNA. Compared with control (siCTL), both protein and mRNA levels of PKD2 were efficiently knocked down by PKD2-siRNA (Fig. 6a). In NCM460 cells, we found that depletion of PKD2 resulted in higher expression of multiple pro-inflammatory cytokines at the mRNA levels (Fig. 6a). We observed a similar effect of PKD2-siRNA on cytokines in Caco-2 cells (Fig. 6b). Moreover, knock-down of PKD2 increased CCL20 secretion in the supernatant of HT-29 cells (Supplementary Fig. S3).

Additionally, we also investigated the impact of different pan-PKD inhibitors on inflammation *in vitro*. Cells were exposed to kb-NB142-70 or CRT0066101 for 24 hours and mRNA expression of pro-inflammatory cytokines were determined by RT-PCR analysis. As shown in Fig. 6c–e, kb-NB142-70 dramatically up-regulated the production of pro-inflammatory cytokines of the 2 cell lines. Similar results were obtained by CRT0066101 treatment (Fig. 6d–f).

Taken together, these data indicate that PKD2 is a potent inhibitor of pro-inflammatory cytokines in colonic epithelial cells, being consistent with the markedly increased colonic mRNA levels of pro-inflammatory cytokines *in vivo* in PKD2 enzymatic deficiency mice.

### PKD2 enzymatic deficiency has minor influence on the B-cell subtypes and the production of serum immunoglobulin

**Figure 7:**
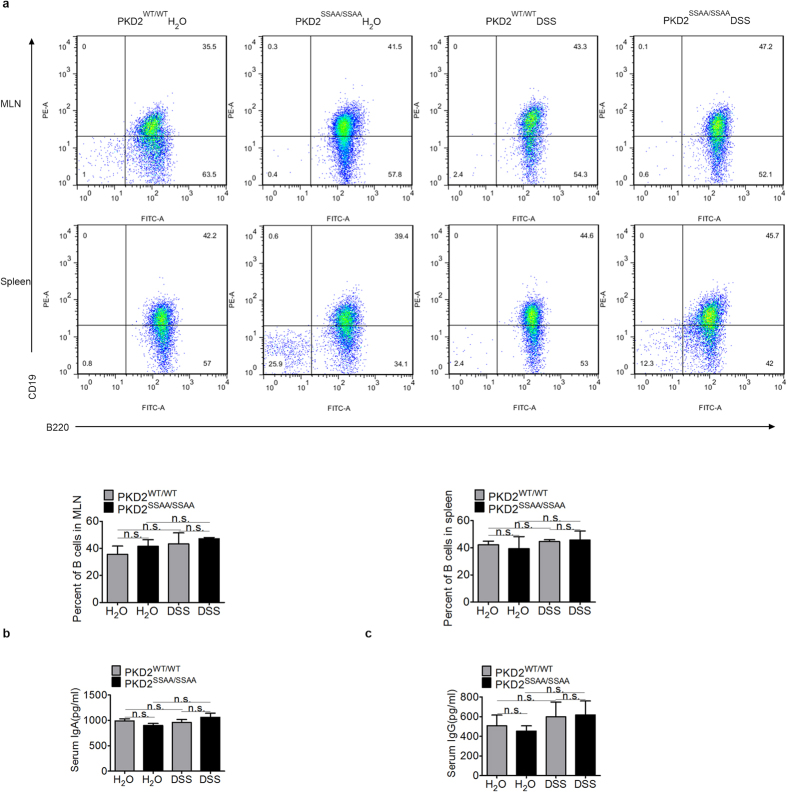
PKD2 effects on the B-cell subtypes and the production of serum immunoglobulin. (**a**) Flow cytometric analysis of B-cell subtypes in MLNs and spleens of PKD2^WT/WT^ and PKD2^SSAA/SSAA^ mice. Total MLNs and splenocytes from PKD2^WT/WT^ and PKD2^SSAA/SSAA^ mice were stained with CD19 and B220. Plots shown are from three representative experiments, and the mean values are indicated. Numbers in the upper plots refer to percentage of CD19^+^ B220^+^ cells. (**b**,**c**) Ig A (**b**) and Ig G (**c**) levels in the serum of mice exposed to DSS were determined by ELISA. Results are mean ± SEM for triplicate samples and are representative of three separate experiments. All p > 0.05 as measured by one way-ANOVA among four groups.

There is evidence demonstrating that B lymphocytes in patients with IBD are hyperactive and produce copious amounts of inflammatory cytokines correlating with increased disease activity^19^,^20^. Thus we investigated the effect of PKD2 enzymatic deficiency on the frequency and function of B cell subsets in mice. Lymphocytes from isolated spleen and MLN in mice following 7 days of DSS were obtained as described below. As shown in Fig. 7a, flow cytometric analysis of mentioned lymphocytes revealed no significant difference in the percentage of B cell (CD19^+^ B220^+^) between PKD2^SSAA/SSAA^ mice and wide-type controls. Additionally, the expression levels of serum IgA (Fig. 7b) and IgG (Fig. 7c) also showed no change between the four groups, suggesting that PKD2 enzymatic deficiency had little effect on the function of B-cell subtypes in acute DSS-induced colitis.

### Down-regulated PKD2 expression in colonic epithelial cells of patients with UC

**Figure 8:**
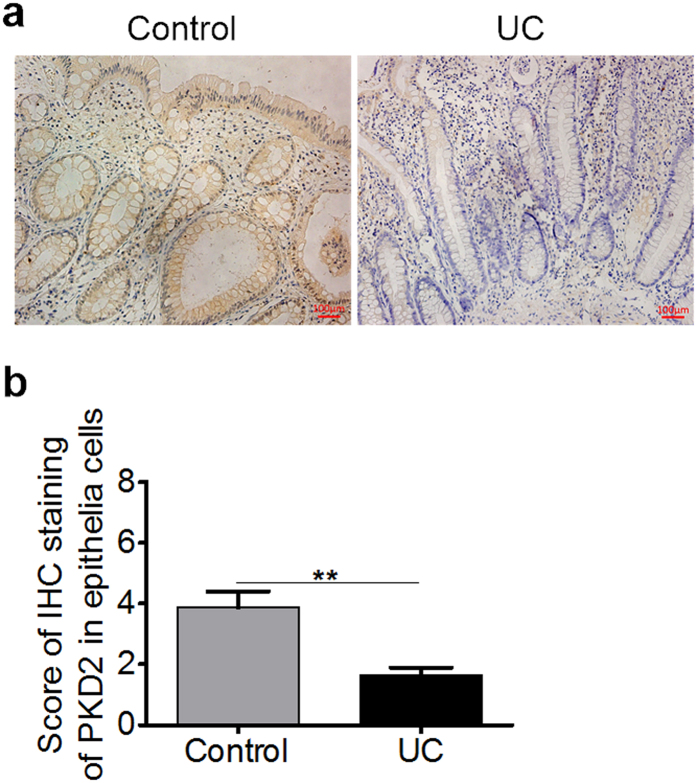
Expression of PKD2 is down-regulated during inflammation. (**a**) Representative micrographs of colonic mucosal biopsies from patients with active UC (right panel) and normal tissues (Control, left panel), stained with anti-PKD2 antibody. Bar represents 100 μm. Images acquired at 200×. (**b**) Immunohistochemistry of PKD2 in epithelial cells was scored from colonic tissue samples of individual patients with UC and normal controls (n = 20).

To verify the possible involvement of PKD2 in human IBD, we performed immunohistochemical analyses of PKD2 in 20 human active UC and 20 normal colonic tissue sections. As shown in Fig. 8a,b, PKD2 staining was observed in the cytoplasm of colonic epithelial cell. More importantly, PKD2 immunostaining in colonic epithelial cells of tissues with active UC was markedly decreased compared with those in cells of normal tissues.

## Discussion

IBD is a chronic, relapsing, destructive and disabling condition. Therapy for IBD has changed remarkably in recent years with the use of biologics, such as anti-tumor necrosis factor (TNF) agents. However, this approach is encountered with increased risk of opportunistic infections^21^,^22^ and a high rate of loss of response to therapy^23^ in patients with IBD, highlighting an urgent need for novel therapeutics for this condition. Emerging studies have shown consistent evidence of an association between PKD2 and inflammatory responses^24^,^25^,^26^. Particularly, PKD2 was found to be the major PKD isoform expressed in murine lymphoid tissues and cells^16^. Based on these findings, we investigated the role of PKD2 in IBD with DSS-induced murine colitis model, which resembled human IBD in the aspect of barrier dysfunction and inflammatory response. Surprisingly, our findings revealed that PKD2 enzymatic deficiency increased susceptibility of mice to DSS-induced colitis. This was associated with an impaired epithelial barrier function and a significant increase in colonic cytokine levels in PKD2 enzymatic deficiency mice compared to wild-type controls, which suggested that PKD2 enzymatic deficiency may contribute to exacerbated inflammation in response to DSS treatment. Hence, our studies provide an insight into a novel role of PKD2 in the suppression of intestinal inflammation by regulating intestinal epithelial permeability and mucosal immunity.

Cytokines have a crucial role in the pathogenesis of IBD. Here, we found that cytokines IL-13, IL-17f and IL-21 were up-regulated in colons of PKD2 enzymatic deficiency mice under physiologic conditions. Moreover, PKD2 enzymatic deficiency mice produced higher levels of IL-1β and IL-6 in response to DSS than wild-type controls. Consistent with this, we observed increased expression of multiple pro-inflammatory genes, such as TNF-α, IL-1β, IFN-γ, IL-8, IL-17a and CCL20 in Caco-2 and NCM460 cells treated with PKD2-siRNA or PKD specific inhibitor *in vitro*. So PKD2 may regulate intestinal mucosal immunity by suppressing the expression of pro-inflammatory cytokines.

Mucus secretion and mucus layer formation are the first line of intestinal mucosal barrier, as evidences demonstrate that Muc2-deficient mice develop spontaneous colitis with depleted mucus layer and elevated levels of pro-inflammatory cytokines^27^,^28^. Intestinal tight junction is mainly comprised of multiple proteins including ZO proteins, occludins and claudins^29^. We have observed down-regulation of ZO-1 and MUC-2 in the colon of PKD2 enzymatic deficiency mice and PKD2-knocked down intestinal epithelial cells and dysfunction of the epithelial barrier confirmed by permeability assay. Furthermore, we found that the barrier-protective effects of PKD2 are associated with tight junctions but not with decreased proliferation or increased apoptosis of intestinal epithelial cells. Similarly, ZO-1, Occludin and MUC-2 were decreased in epithelial cells treated with different PKD specific inhibitors, which could explain the increased intestinal epithelial permeability in Caco-2 cells.

A number of investigators have reported that several pro-inflammatory cytokines, such as TNF-α, IL-1β and IFN-γ, have been shown to increase intestinal epithelial permeability by modulating tight junction protein expression and cellular localization^30^,^31^,^32^,^33^,^34^. As described above, the observed elevation of cytokine expression by PKD inhibition may therefore represent a potential mechanism for barrier disruption with an increased permeability *in vitro*.

In addition to aberrant T cells response, intestinal B cells in patients with IBD are increased^35^ and their antibody-secreting function enables B cells to be part of the immune regulation and homeostasis^36^. Also in our experimental colitis models, the frequency and antibody-secreting function of B cells were detected, while no differences were observed. It thus appears that PKD2 may have no effect on B cells in the context of colitis.

There is increasing evidence demonstrating that PKD family play a pivotal role in inflammatory response^37^,^38^ and barrier function^39^. Hao Q *et al*. reported that silencing PKD2 in endothelial cells markedly inhibited the production of pro-inflammatory cytokine IL-6, IL-8 and GRO-α by VEGF^40^. Another study showed that cytokine-induced disruption of pancreatic ductal epithelial barrier was accompanied by JAK and PKD dependent decrease in expression of junction protein^41^. Surprisingly, our results differ from their observations. More importantly, our findings provide unequivocal genetic evidence *in vitro* and *in vivo* that PKD2 protects against DSS-induced colitis by regulating inflammation and epithelial barrier function. At present, the precise mechanisms by which PKD2 modulates immune response and epithelial barrier function are still unclear. Another study demonstrated that myeloid-restricted inactivation of PKD1 resulted in exacerbated lung inflammation by the mechanism that PKD1 phosphorylated p85α to enhance its interaction with PTEN, thereby repressed neutrophil migration^42^. As PKD1 and PKD2 shares 85% similarities in amino acid level, their regulatory mechanism in most of the cells are the same. Hence, further identification of PKD2-involved pathways and molecular targets will be essential in understanding the mechanisms by which PKD2 affects the pathogenesis of intestinal inflammation.

In summary, our present studies demonstrate for the first time that PKD2 exerts a protective effect on experimental colitis, highlighting the relevance of PKD2 as a potential therapeutic molecule to treat IBD.

## Methods

### Mice

Mice used in the study were housed in specific pathogen-free conditions and allowed free access to standard food and tap water. All animal protocols were approved by the Institutional Animal Care and Use Committees of Southern Medical University and were carried out in accordance with the relevant guidelines.

PKD2^S707A/S711A^-knockin mice (heterozygous PKD2 catalytic activity deficiency mutant mice) on a C57BL/6 background were originally obtained from the Jackson Laboratory (Bar Harbor, ME). Homozygous PKD2 catalytic activity deficiency mutant mice (PKD2^SSAA/SSAA^) were obtained by intercrossing heterozygous mice (PKD2^WT/SSAA^).

### DSS-induced colitis and Histology

Mice used in this study were six-eight week-old homozygous PKD2 catalytic activity deficiency mice (PKD2^SSAA/SSAA^). Control C57BL/6 mice (PKD2^WT/WT^) were matched for age and gender in each experiment. Eight mice were included in each group. Colitis was induced in mice by drinking water supplemented with 5% DSS (MW = 36,000–50,000, MP Biomedicals, California, USA) for 7 days^43^, control mice received tap water without DSS. Body weight change, stool consistency and gross bleeding were assessed daily. The disease activity index (DAI) score was calculated, as previously described^44^. At day 7, mice were sacrificed by cardiac puncture (under pentobarbital sodium anesthesia) and the colon was removed for subsequent analysis. Colon samples for histological analyses were fixed in 4% paraformaldehyde and stained with hematoxylin and eosin (H&E). The colitis was analyzed and scored by two blinded pathologists according to a previous study^45^.

### Cell lines, cell culture and siRNA transfection

Caco-2 (human intestinal epithelial adenocarcinoma cells) were obtained from ATCC and were grown in DMEM (Gibco, Carlsbad, CA) supplemented with 15% fetal bovine serum at 37 °C in a humidified 5% CO2 atmosphere. NCM460 (normal human colon epithelial cells) were purchased from INCELL Corporation (San Antonio, TX, USA) and maintained in M3:10 media supplemented with 10% FBS at 37 °C in a humidified 5% CO2 atmosphere. The small interfering RNAs (siRNAs) were transfected into cells using Lipofectamine 3000 according to the manufacturer’s instructions. The following siRNA sequences were used (GenePharma, Shanghai, China): PKD2-Forward, 5′-CCUGAGUGUGGCUUCUACGGCCUU-3′, PKD2-Reverse, 5′-AAAGGCCGUAGAAGCCACACUCAG-3′.

The cells were used 2 or 3 days after transfection for western blot and messenger RNA (mRNA) analysis.

### Western blot analysis

Cells or colonic tissues were lysed in lysis buffer composited of 100 mM Tris-HCl, 200 mM DTT, 4% SDS, 20% glycerol and 0.004% bromophenol blue (all reagents were obtained from Sigma). Proteins were analyzed by SDS-PAGE and transferred onto nitrocellulose membranes (Bio-Rad). Membranes were blocked and incubated overnight at 4 °C with the indicated antibodies and proteins were then detected by the enhanced chemiluminescence method (SuperSignal West Pico substrate, Pierce, Rockford, IL). α-tubulin was used as a loading control for all western blot assays. Antibodies used for western blot analysis are α-tubulin (Catalog #RM2007L, Beijing Ray Antibody Biotech, China), phospho-PKD (Ser916) (Catalog #2051), phospho-PKD (Ser744/748) (Catalog #2054), PKD1 (Catalog #2052), PKD2 (Catalog #8188) and PKD3 (Catalog #5655) (Cell Signaling Technology, Danvers, MA), ZO-1 (Catalog #40-2200) and Occludin (Catalog #710192) (Invitrogen), MUC2 (Catalog #5251-1, Epitomics).

### RNA extraction and real time quantitative RT-PCR analysis

Total RNA was isolated from colon of mice or cells using Trizol reagent (Invitrogen) following the manufacturer’s protocol. Reverse transcription and RT-PCR analysis were carried out using the All-in-One First-Strand cDNA Synthesis Kit and All-in-One qPCR Mix (GeneCopoeia) according to the manufacturer’s protocol. UBC was used as a loading control for all RT-PCR. All primers for RT-PCR were purchased from Invitrogen Life Technologies and sequences of primers are listed in Supplementary Table S1.

### *In Vitro* Permeability Assay

1 × 10^5^ Caco-2 cells were seeded on 0.4 μm porous Transwell polycarbonate membranes (Transwell 3401; Corning, NY, USA) in 12-well plates. Then 0.5 and 1 mL of culture medium were added to the upper and lower chambers, respectively. The Transwell plates were then incubated at 37 °C in a humidified 5% CO_2_ atmosphere. The culture media was changed every other day. When cells reached confluence (18–21 days after seeding), cells were exposed to PKD specific inhibitor CRT0066101 (5 μM, Catalog #SML1507, Sigma-Aldrich, St. Louis, MO, USA) or kb-NB142-70 (10 μM, Catalog #3962, TOCRIS bioscience) dissolved in DMSO for up to 24 hours. Following inhibitors treatment, 1 mg/mL paracellular marker fluorescein isothiocyanate (FITC)-dextran (4 kDa) was added to the apical well of each insert and the monolayer was incubated for 2 hours at 37 °C. Monolayer permeability to FITC-dextran was then determined by measuring the fluorescence level in the bottom chamber at 485 nm (excitation wavelength) and 535 nm (emission wavelength). Values were converted in concentrations of FITC-dextran (pg/mL) using a standard curve. Three independent experiments were performed.

### *In Vivo* Permeability Assay

Mice were gavaged with 4 kDa FITC-dextran (0.6 mg/g body weight) after exposing to 6 days of DSS treatment, then sacrificed 4 hours later and their plasma was separated for fluorescence measurements. The distribution of FITC-dextran in frozen sectioned colonic tissue was determined by fluorescence microscopy as well.

### Flow Cytometry

Single-cell suspensions of murine spleen and mesenteric lymph nodes (MLN) were obtained using the mouse lymphocyte separation medium (Dakewe Biotech Company, Limited, China)^46^ according to manufacturer instructions and stained with anti-mouse CD19 phycoerythrin (PE) (Catalog #12-0193-81, eBioscience, San Diego, CA) and anti-mouse CD45R/B220 flurorescein isothiocyanate (FITC) (Catalog #11-0451-81, Bioscience, San Diego, CA) according to the manufacturers’ instructions. Data were acquired using FACSCalibur with CellQuest software (BD Biosciences, San Jose, CA) and analyzed using FlowJo 7.6.1 (Tree Star, Ashland, OR).

### ELISA

Blood was withdrawn by cardiac puncture of anesthetized mice. Concentrations of immunoglobulin A (IgA) and immunoglobulin G (IgG) in serum of mice were measured using the mouse ELISA kit (Elabscience Biotechnology), according to the manufacturer’s instructions.

### Immunohistochemistry

Colonic mucosal biopsies were obtained from 20 patients with active UC who had undergone colonoscopy examination at Nanfang Hospital, Guangzhou, China. These patients’ conditions were diagnosed by certified hospital gastroenterologists. Disease activity of patients was assessed with the Modified Mayo Clinic Score consisting of 4 criteria: stool frequency, rectal bleeding, colonoscopy findings, and assessment of patients’ function. Each diagnosis is graded on a scale of 0 to 3.Total grade <2 indicates remission, grade of 3–5 indicates mildly active, 6–10 indicates moderately active and 11–12 indicates severely active UC. Among the 20 patients, 7 have mildly active UC and 13 others have moderately active UC. Tissue samples from patients who have normal mucosa according to endoscopic and histological criteria were used as normal control. A pathologist confirmed all the histological diagnoses. Written informed consent was obtained from all subjects. All experimental protocols were approved by the Ethics Committee of Nanfang Hospital affiliated to Southern Medical University and were carried out in accordance with the approved guidelines.

Immunohistochemical staining of PKD2 (Catalog #ENT3773, Elabscience) was graded independently for both the extent and intensity of immunopositivity by two experienced pathologists in a blinded fashion as described by Nagai *et al*.^47^. In the case of Ki-67 (Catalog #NB500-170, Novus Biologicals) and cleaved Caspase-3 (Catalog #9664, Cell Signaling Technology) for each section, three 200× fields, were randomly selected, and the number of positive cells per 100 intestinal epithelial cells was quantified.

### Statistics

Data were expressed as mean ± SEM and all the statistical analyses were performed by SPSS 21.0 software (SPSS, Chicago, IL) using analysis of variance (ANOVA) or t test where appropriate. P < 0.05 was considered statistically significant and levels of significance were assigned as *p < 0.05, **p < 0.01, ***p < 0.001.

## Additional Information

**How to cite this article**: Xiong, J. *et al*. Protein Kinase D2 Protects against Acute Colitis Induced by Dextran Sulfate Sodium in Mice. *Sci. Rep.* **6**, 34079; doi: 10.1038/srep34079 (2016).

## Supplementary Material

Supplementary Information
