# Supplementary material for: Protein Kinase D2 Protects against Acute Colitis Induced by Dextran Sulfate Sodium in Mice
Source: Sci Rep. 2016 Sep 23;6:34079. doi: 10.1038/srep34079 (PMC5034322; doi:10.1038/srep34079)
Supplement: Supplementary Information [file srep34079-s1.pdf]

Protein Kinase D2 Protects against Acute Colitis Induced by Dextran Sulfate Sodium in Mice

Jing Xiong[1], Ming-feng Zhou[1,2], Ya-dong Wang[1], Li-ping Chen[2], Wan-fu Xu[2], Yao-dong Wang[2], Fan Deng[2*], Si-de Liu[1*]

[1]Guangdong Provincial Key Laboratory of Gastroenterology, Department of Gastroenterology, Nanfang Hospital, Southern Medical University, Guangzhou 510515, China

[2]Department of Cell Biology, School of Basic Medical Sciences, Southern Medical University, Guangzhou 510515, China

Supplementary Figures and Table

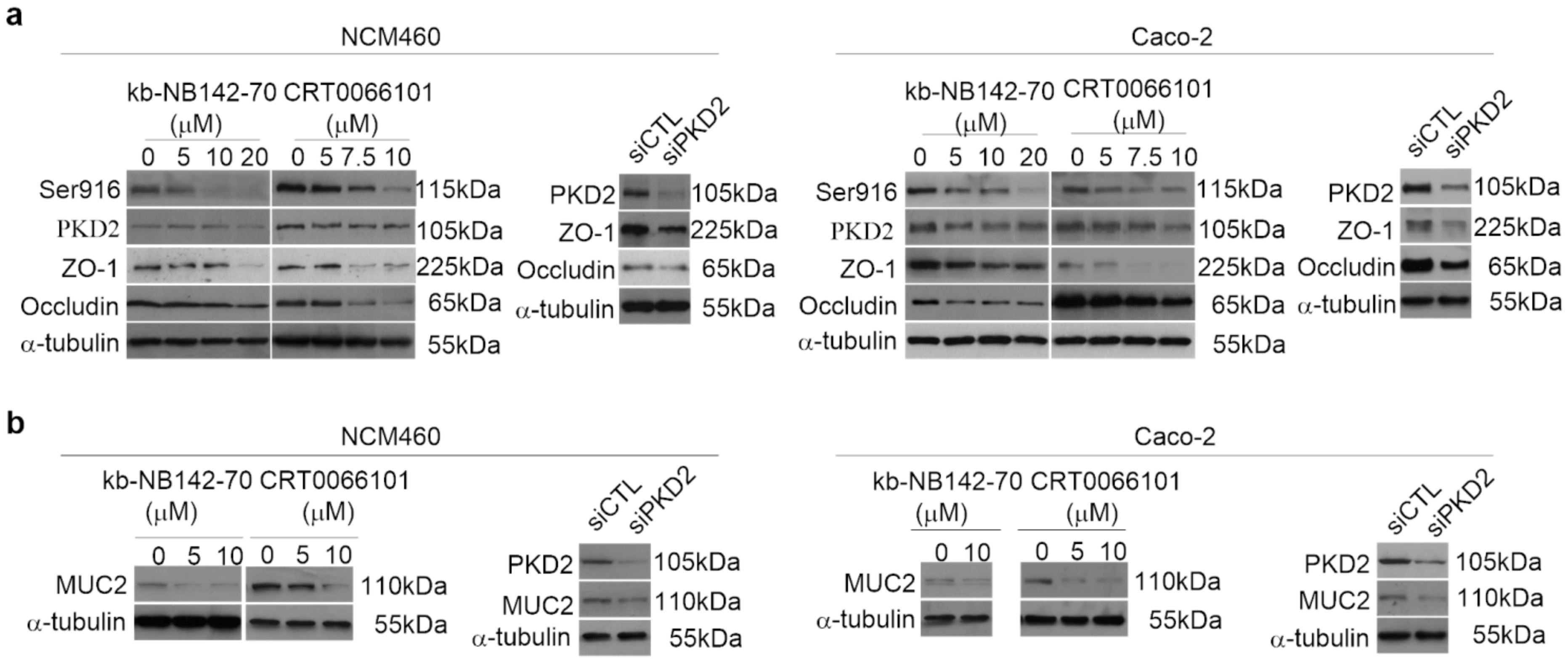

Figure S1. Inhibition of PKD2 activity or silencing of PKD2 in vitro both down-regulated TJs and MUC2 of intestinal epithelial cell monolayers. Inhibition of PKD2 activity or silencing of PKD2 both down-regulated TJs (a) and MUC2 (b) of intestinal epithelial cell monolayers.

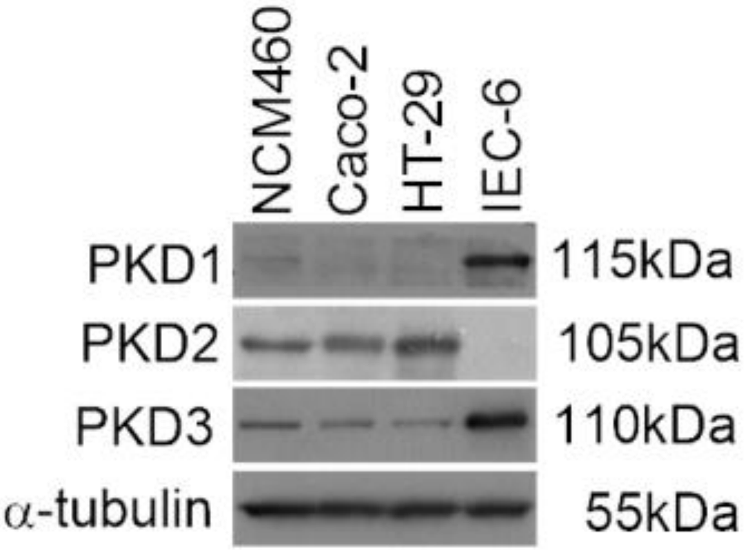

Figure S2. **Expression of PKD in different colon epithelial cell lines.** Normal colon epithelial cells and colorectal cancer cells were harvested and processed for western blot analysis.

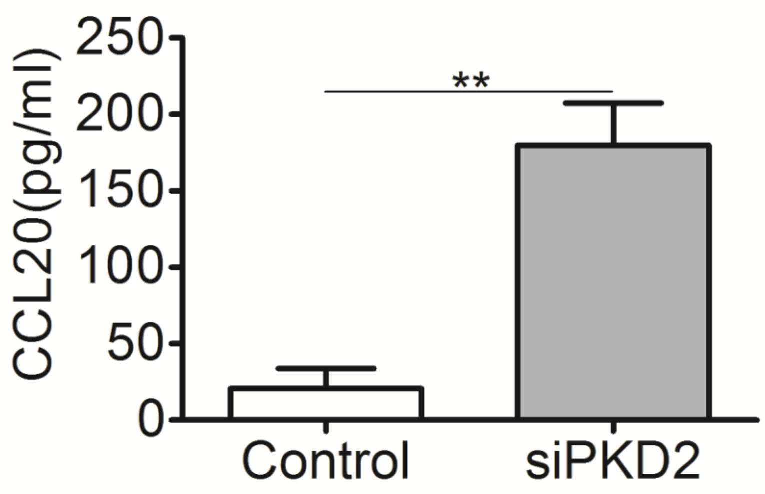

Figure S3. Silencing of PKD2 in HT-29 cells increased the secretion of CCL20 in the supernatant. HT-29 cells were transiently transfected with PKD2 siRNA (siPKD2). At 60 hours post-transfection, the supernatant was collected and the concentration of CCL20 was determined by ELISA. Results are mean±SEM for triplicate samples and are representative of three separate experiments.

Table S1. Sequences of the amplification primers used in this study

Sequence Name	Sequence (5' to 3')
Mus GAPDH-F	GGACCTCATGGCCTACATGG
Mus GAPDH-R	TAGGGCCTCTCTTGCTCAGT
Mus IL-13-F	CCTGGCTCTTGCTTGCCTT
Mus IL-13-R	GGTCTTGTGTGATGTTGCTCA
Mus IL-17f-F	TGCTACTGTTGATGTTGGGAC
Mus IL-17f-R	AATGCCCTGGTTTTGGTTGAA
Mus IL-21-F	GGACCCTTGTCTGTCTGGTAG
Mus IL-21-R	TGTGGAGCTGATAGAAGTTCAGG
Mus IL-1β-F	GCAACTGTTCCTGAACTCAACT
Mus IL-1β-R	ATCTTTTGGGGTCCGTCAACT
Mus IL-6-F	TAGTCCTTCCTACCCCAATTTCC
Mus IL-6-R	TTGGTCCTTAGCCACTCCTTC
Homo UBC-F	ATTTGGGTCGCGGTTCTTG
Homo UBC-R	TGCCTTGACATTCTCGATGGT
Homo si-PKD2-F	CCUGAGUGUGGCUUCUACGGCCUU

Homo si-PKD2-R	AAAGGCCGUAGAAGCCACACUCAG
Homo TNF-α-F	CCTCTCTCTAATCAGCCCTCTG
Homo TNF-α-R	GAGGACCTGGGAGTAGATGAG
Homo IL-1β-F	TTCGACACATGGGATAACGAGG
Homo IL-1β-R	TTTTTGCTGTGAGTCCCGGAG
Homo IFN-γ-F	TCGGTAACTGACTTGAATGTCCA
Homo IFN-γ-R	TCGCTTCCCTGTTTTAGCTGC
Homo IL-8-F	TTTTGCCAAGGAGTGCTAAAGA
Homo IL-8-R	AACCCTCTGCACCCAGTTTTC
Homo IL-17a-F	TCCCACGAAATCCAGGATGC
Homo IL-17a-R	GGATGTTCAGGTTGACCATCAC
Homo CCL20-F	TGCTGTACCAAGAGTTTGCTC
Homo CCL20-R	CGCACACAGACAACTTTTTCTTT
